# Increased PKMζ activity impedes lateral movement of GluA2-containing AMPA receptors

**DOI:** 10.1186/s13041-017-0334-7

**Published:** 2017-11-29

**Authors:** Nam-Kyung Yu, Heesoo Uhm, Jaehoon Shim, Jun-Hyeok Choi, Sangsu Bae, Todd Charlton Sacktor, Sungchul Hohng, Bong-Kiun Kaang

**Affiliations:** 1 Department of Biological Sciences, College of Natural Sciences, Seoul National University, Seoul, South Korea; 2 Department of Physics and Astronomy, Seoul National University, Seoul, South Korea; 3 National Center for Creative Research Initiatives, Seoul National University, Seoul, South Korea; 4 Institute of Applied Physics, Seoul National University, Seoul, South Korea; 5 Department of Biophysics and Chemical Biology, Seoul National University, Seoul, South Korea; 6 Institute of Nano Science and Technology, Hanyang University, Seoul, South Korea; 7 Department of Physiology & Pharmacology, SUNY Downstate Medical Center, 450 Clarkson Ave, Brooklyn, NY 11203 USA; 8 Department of Anesthesiology, SUNY Downstate Medical Center, 450 Clarkson Ave, Brooklyn, NY 11203 USA; 9 Department of Neurology, SUNY Downstate Medical Center, 450 Clarkson Ave, Brooklyn, NY 11203 USA; 10 Center for Neuron and Disease, Frontier Institute of Life Science and of Science and Technology, Xi’an Jiaotong University, Xi’an, China

**Keywords:** PKMζ, PKM-zeta, AMPAR, Quantum dots, Single molecule imaging, GluA2, LTP, Lateral diffusion

## Abstract

Protein kinase M zeta (PKMζ), a constitutively active, atypical protein kinase C isoform, maintains a high level of expression in the brain after the induction of learning and long-term potentiation (LTP). Further, its overexpression enhances long-term memory and LTP. Thus, multiple lines of evidence suggest a significant role for persistently elevated PKMζ levels in long-term memory. The molecular mechanisms of how synaptic properties are regulated by the increase in PKMζ, however, are still largely unknown. The α-amino-3-hydroxy-5-methyl-4-isoxazolepropionic acid (AMPA) receptor (AMPAR) mediates most of the fast glutamatergic synaptic transmission in the brain and is known to be critical for the expression of synaptic plasticity and memory. Importance of AMPAR trafficking has been implicated in PKMζ-mediated cellular processes, but the detailed mechanisms, particularly in terms of regulation of AMPAR lateral movement, are not well understood. In the current study, using a single-molecule live imaging technique, we report that the overexpression of PKMζ in hippocampal neurons immobilized GluA2-containing AMPARs, highlighting a potential novel mechanism by which PKMζ may regulate memory and synaptic plasticity.

## Introduction

Long-term memory and its cellular analog long-term potentiation (LTP), undergo induction and maintenance processes involving distinct mechanisms [1]. Whereas induction is believed to be regulated by post-translational modification of pre-existing proteins at the synapse, maintenance is thought to require new protein synthesis during brief time windows after learning followed by the persistent activity of newly synthesized proteins such as protein kinase M zeta (PKMζ) [2–4]. PKMζ is produced by internal transcription from the PKCζ gene, lacking the autoinhibitory domain of PKCζ and is thus autonomously active.

Numerous studies have shown that the administration of a PKMζ inhibitor, zeta inhibitory peptide (ZIP), during the maintenance phase erased various types of memory and reversed LTP, suggesting PKMζ as a key molecule for memory maintenance [5–8]. However, this has been challenged by new findings where the genetic deletion of PKMζ in mice did not impair the formation of long-term memory and LTP [9, 10]. Moreover, ZIP still impaired memory and LTP in PKMζ-null mice, questioning the specificity of ZIP. Although the exact mechanisms how ZIP has abolished long-term memory and LTP remain to be sorted out, it has been consistently shown that PKMζ protein level increases after learning or LTP-inducing stimuli [9, 11, 12]. The up-regulation has been reported to last up to 1 month, much longer than for most other proteins known to be induced after learning [12]. In that study, the persistence of the up-regulation was correlated with the persistence of memory. Importantly, PKMζ-specific antisense oligonucleotide that prevents the up-regulation impaired the late-LTP and memory, suggesting the necessity of the up-regulation [11, 12]. Moreover, a recent study demonstrated that another type of atypical protein kinase C, iota/lambda (PKCι/λ), which can be also blocked by ZIP, compensates the PKMζ’s roles for late-LTP and certain types of long-term memory in PKMζ-null mice, whereas in wild-type mice the PKCι/λ antagonist had no apparent effect [11]. In addition, a second study using shRNA to knockdown either PKMζ or PKCι/λ found that suppression of PKMζ, but not PKCι/λ, disrupted previously established long-term memory [13]. Therefore, in normal conditions without compensation due to the prolonged absence of PKMζ, the persistent increase of PKMζ following learning or an LTP stimulus may play a crucial role in the maintenance of memory and LTP. The importance of PKMζ in memory has been further supported by the studies showing that PKMζ overexpression enhances synaptic transmission and plasticity [14] as well as long-term memory [15, 16].

However, the molecular mechanisms underlying how the increased PKMζ contributes to synaptic transmission and memory maintenance are not well understood. The α-amino-3-hydroxy-5-methyl-4-isoxazolepropionic acid (AMPA) receptor (AMPAR) is the major glutamate receptor in the brain that mediates fast synaptic transmission. Its level at the synaptic surface is critical for determining synaptic strength and thus its regulation is crucially involved in not only synaptic plasticity and memory but also disorders such as chronic pain and itch [17–21]. The level of surface AMPARs can be regulated by vesicle trafficking between membrane and intracellular compartments via exocytosis and endocytosis [22, 23]. Previous studies have suggested that PKMζ regulates the synaptic level of AMPA receptor by promoting the interaction of GluA2-containing AMPARs with NSF and disrupting the internalization of AMPAR via PICK1/AP2 [24, 25]. In addition to membrane trafficking, the lateral movement of AMPAR on the cell membrane and its diffusional trapping at synapses have emerged as central mechanisms determining the distribution of AMPAR [24]. However, whether PKMζ can act on the lateral mobility of membrane AMPAR has not been explored.

In the current report, therefore, we used single quantum-dot (Qdot) live-imaging to examine the possibility that PKMζ regulates the lateral diffusion of AMPARs.

## Methods

### Dissociated rat hippocampal neuron culture and nucleofection

The culture was prepared by following the protocol from our previous study [26] with modifications for nucleofection and imaging. A Lab-tek II 4 chamber slide glass (Nunc, 154,534) was first coated with poly-D-lysine in sodium borate buffer (pH 8.5), followed by laminin. Dissociated hippocampal cells from E18 rat embryos were mixed with DNA constructs (330 ng of each construct) in the strips for nucleofection, following the manufacturer’s manual (Lonza). For DNA constructs, pZcb-rtTA3 and pZtiwb-mCerulean3.0-homer1c were included in every group, and either pZtiwb-PKM**ζ** (K98W), pZtiwb-PKM**ζ** (T227E), or no PKMζ (as a control) were added. The nucleofected and non-nucleofected cells were mixed, at a ratio of 2:1, and plated at density of 18,000 cells/cm^2^ in the plating media. After 3 h of recovery, media were changed into maintenance media.

### Labeling AMPARs

Neurons at 13–14 DIV were treated with doxycycline (0.7 μg/mL) for 2 days to induce the expression of PKM**ζ** mutants and mCerulean3.0-homer1c, under the tetO promoter. Following a 10-min incubation with anti-GluA2 antibody (MAB397, 1:200) in the imaging buffer (124 mM NaCl, 2.5 mM KCl, 1 mM NaH_2_PO_4_, 2 mM CaCl_2_, 2 mM MgCl_2_, 24.6 mM NaHCO_3_, 30 mM D-glucose, 20 mM HEPES, pH 7.4), the neurons were washed with the imaging buffer twice and incubated with Qdot655 anti-mouse IgG (H + L) for 2 min (Invitrogen, Q-11021MP, 1:200). The neurons were then washed with the imaging buffer twice and placed under the microscope for single molecule imaging within 30 min after labeling.

### Single molecule localization and tracking

Neurons were imaged at 37 °C using an inverted microscope (Olympus IX71, Olympus), equipped with an electron multiplying charge-coupled device camera (Ixon DV897, Andor Technology). The mCerulean3.0-Homer1C and Qdot (Qdot655, Thermo Fisher)-AMPAR were excited using blue (473 nm, Blues TM50, Cobolt) and red lasers (640 nm, Cube640-100C, Coherent), respectively. Secondary dendrites (at most 3 different regions per cell) were randomly selected for imaging. A total of 100 consecutive frames were first imaged for mCerulean3.0-Homer1C, while 500 frames were recorded later for Qdot-AMPAR, at 20 Hz (with an exposure of 50 ms). The same protocol was repeated at different positions. The total imaging time for a sample was limited to less than 30 min. The localization and tracking of single Qdots were performed using homemade programs written in MATLAB (Mathworks). To determine the center position, single Qdot images were fitted to a two-dimensional Gaussian surface [27]. When continuous imaging of the Qdot was not possible, due to Qdot blinking, the trajectories of the same Qdot were connected using criteria that the maximal dark period of Qdot was less than 10 frames (i.e., 0.5 s) and the maximal positional change was less than 4 pixels (i.e., 280 nm) [28]. Synapses were identified using image segmentation of the mCerulean3.0-Homer1C postsynaptic marker, and the corresponding binary mask was then used to sort Qdot-AMPAR trajectories to synaptic or extrasynaptic regions. Diffusion coefficients were calculated using a linear fit of the first 4 points of the mean square displacement (MSD) plots versus time. Immobile fractions of the trajectories were determined using the criteria that the logarithm of the diffusion coefficient (in units of μm^2^ s^−1^) was less than −2.5.

### Immunocytochemistry

The neurons expressing PKMζ T227E or K98W under the same condition used for the Qdot live imaging were fixed using 4% paraformaldehyde/4% sucrose in PBS for 15 min at RT and washed with PBS for 3 times. Cells were permeabilized by PBT (0.1% BSA, 0.1% Triton X-100 in PBS) at RT for 15 min. After blocking at RT for 2 h in blocking solution (2% BSA, 0.08% Triton X-100 in PBS), antibody (rabbit anti-PKCζ, Cell Signaling #9368, 1:500) was incubated at 4 °C o/n. After washing twice with PBT at RT for 15 min, cells were incubated with secondary antibody (ALEXA 647 anti-rabbit IgG, Invitrogen #A31573, 1:500) at RT for 2 h and then washed with PBT and PBS. Images were taken by confocal microscope (LSM700).

### Electrophysiology

For whole-cell patch-clamp recordings, rat cultured hippocampal neurons at DIV 13–14 were used for patch clamp recording. Neurons were voltage clamped at −70 mV for mEPSC recording, using a Multiclamp 700B amplifier and pClamp 10.4 software (Molecular Devices). Data were collected for 5 min and digitized at 10 kHz, with a 2-kHz lowpass filter, and dizitized by Digidata 1440 16-bit A/D converter (Axon instruments). Recording pipettes (3 ~ 4.5 MΩ) were pulled with P-1000 (Sutter instrument) using a four-step protocol. First the pipettes were filled with internal solution containing the following: 145 mM K-Gluconate, 5 mM NaCl, 0.2 mM EGTA, 10 mM HEPES, 2 mM MgATP, 0.1 mM Na_3_GTP, and 1 mM MgCl2 (pH 7.2 with KOH, 280 ~ 290 mOsm). For mEPSC recording, picrotoxin (100 μM) and tetrodotoxin (1 μM) were added to the external solution containing the following: 140 mM NaCl, 3 mM KCl, 10 mM Glucose, 10 mM HEPES, 2 mM CaCl_2_, 2 mM MgCl_2_. We excluded the data if its series resistant significant changes (> 20%) or it reached 15 MΩ. Cells that need more than 200 pA of hold current to maintain −70 mV were also excluded from the dataset. Data were analyzed using Clampfit 10.5 (Molecular Devices) with template match function with threshold of 3 by creating template using 50 sample traces in the data. The template was used for analyzing all the data.

## Results and discussion

**Fig. 1:**
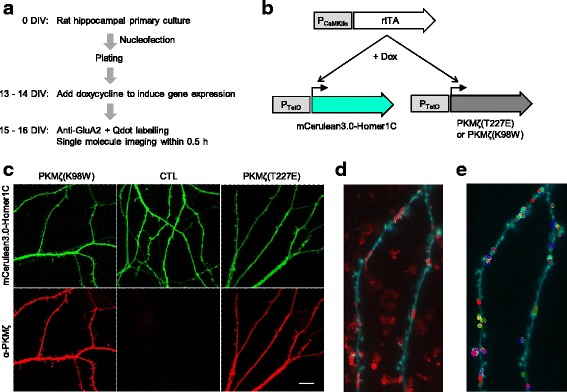
Overexpression of active or inactive PKMζ in cultured rat hippocampal neurons and single molecule imaging of GluA2-containing AMPARs. **a** Experimental workflow. **b** Transgene expression using TetON system. **c** Representative image of fluorescence microscopy of Homer1C-fused mCerulean3.0 and anti-protein kinase C, zeta (PKCζ) immunofluorescence signals in neurons expressing active PKMζ (T227E), inactive PKMζ (K98W), or neither (CTL). **d** Representative image of Qdot-labelled GluA2-containing AMPARs (red, maximum intensity projection of 500 frames) and mCerulean3.0 (cyan, average image of 100 frames). (**e**) Qdot trajectories (open circles) observed in the dendritic region (cyan)

To explore whether PKMζ regulates the lateral movement of AMPARs, we expressed active and inactive PKMζ mutants in cultured rat hippocampal neurons and performed single molecule live imaging of endogenous AMPARs (Fig. 1). PKMζ becomes fully functional through the phosphorylation of T227, which corresponds to T410 in PKCζ, by PDK1 [29]. Therefore, we used the phosphomimetic form (T227E) to assess the effect of elevating PKMζ activity. K98, which corresponds to K281 in PKCζ, is located in the ATP-binding site of PKMζ and is essential for PKMζ kinase activity. The expression of the K98W mutant form of PKMζ has been shown to impair memory maintenance [15, 30] and LTP [5]. We expressed the K98W mutant form of PKMζ to examine the effect of hindered PKMζ activity. Homer1C, a postsynaptic marker, fused with the fluorescent protein mCerulean3.0, was co-expressed for visualizing the transfected neurons and identifying the synaptic regions (Fig. 1b, c). We employed a TetON expression system, which was activated by doxycycline-inducible rtTA; the expression of the system was driven by a neuron-specific CaMKIIα promoter (Fig. 1b). We introduced the plasmids into cells by nucleofection before plating cells and added doxycycline into the culture media at 14–15 DIV. After 2 days of expression, we labelled endogenous surface AMPARs by brief incubation of neurons with a primary antibody against GluA2, which was followed by the incubation with a secondary antibody linked to Qdots [28]. We focused on GluA2 subunit-containing AMPARs because its synaptic incorporation is thought to be important for LTP and long-term memory maintenance [31–33].

**Fig. 2:**
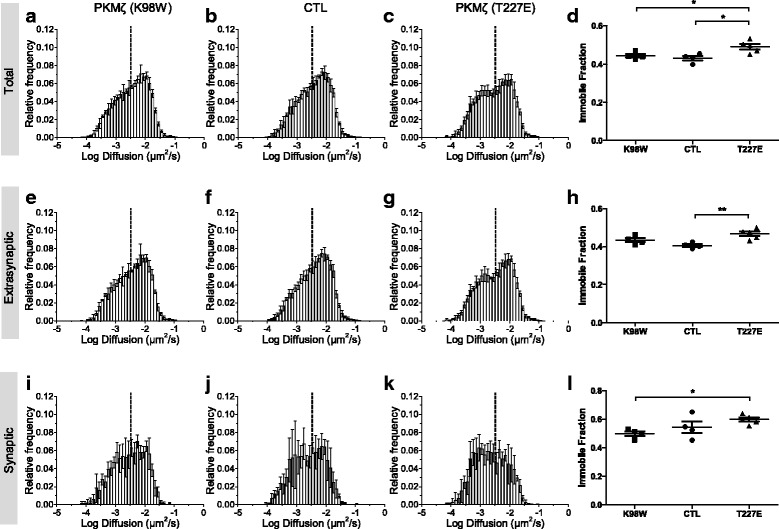
The overexpression of PKMζ increases the immobile fraction of GluA2-containing AMPARs in both extrasynaptic and synaptic regions. The relative frequency distribution of diffusion coefficients of Qdot-labelled GluA2-containing AMPARs in neurons expressing PKMζ K98W (total: **a**, extrasynaptic: **e**, synaptic: **i**), T227E (total: **c**, extrasynaptic: **g**, synaptic: **k**), or control neurons (CTL) expressing mCerulean3.0-Homer1C alone (total: **b**, extrasynaptic: **f**, synaptic: **j**). Data are represented as mean ± standard deviation (S.D.) (**d**, **h**, **l**) Fraction of immobile receptors using the threshold in each set of experiment. Data were analyzed using a one-way analysis of variance (ANOVA), with post-hoc Newman Keuls multiple comparison test (* *p* < 0.05, ** *p* < 0.01). Data are represented as mean ± standard error of mean (S.E.M.)

We used a home-made program (see Methods) to measure the diffusion rates of AMPARs in an unbiased and automatic way. The relative distribution of diffusion coefficients of Qdot-labelled GluA2-containing AMPARs appeared similar to those that were obtained from a large number of AMPARs using super-resolution imaging [34]. T227E expressing neurons showed higher fraction of immobile GluA2-containing receptors, compared with control or K98W neurons, suggesting that the increase of PKMζ activity restrains the lateral movement of GluA2-containing AMPARs (Fig. 2a–d).

Next, to elucidate subcellular locations where the changes in AMPAR movement occurred, we classified the trajectories into synaptic or extrasynaptic trajectories based on the Homer1C-fused mCerulean3.0 fluorescence intensity (see Methods). In our analysis, T227E neurons showed an increase in GluA2 immobile fraction in both extrasynaptic and synaptic regions (Fig. 2e–l), suggesting that PKMζ affected the machinery involved in the lateral movement of GluA2-containing AMPARs, regardless of their synaptic or extrasynaptic localization.

**Fig. 3:**
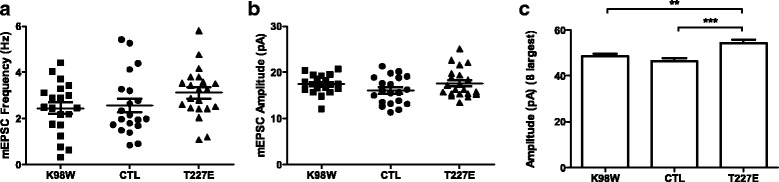
Overexpression of PKMζ augments the size of large-amplitude miniature excitatory postsynaptic currents (mEPSCs). **a** The mEPSC frequency of neurons expressing PKMζ K98W, PKMζ T227E, or control neurons at DIV 16–17 (One-way ANOVA, *p* = 0.1697, *n* = 20–21 neurons). **b** The mean mEPSC amplitude of neurons expressing PKMζ K98W, PKMζ T227E, or control neurons at DIV 16–17 (One-way ANOVA, *p* = 0.1483). **c** The amplitude of 8 largest mEPSCs from each cell (PKMζ K98W, n = 20 neurons, *n* = 160 events; Control, *n* = 20 neurons, *n* = 160 events; PKMζ T227E, *n* = 21 neurons, *n* = 168 events; One-way ANOVA with post-hoc Newman Keuls multiple comparison test (**p < 0.01, ***p < 0.001)). Data are represented as mean ± S.E.M.

Next, we recorded the miniature excitatory synaptic transmission current (mEPSC) in these neurons to investigate whether the suppression of the lateral movement of AMPARs due to PKMζ activity was associated with a change in synaptic transmission. Earlier work by Ron et al. (2013) [35] overexpressing PKMζ in cultured neocortical neurons had found a selective increase in the amplitude of the largest mEPSCs, without a change in the average mEPSC amplitude or frequency. We observed in hippocampal neurons a tendency for an increase in frequency and amplitude of the mEPSCs in PKMζ T227E-transfected neurons although statistically nonsignificant (Fig. 3a, b), and, in line with Ron et al. (2013) [35], that the amplitudes of largest mEPSCs from each cell are higher in the PKMζ T227E group as compared with the control and K98W neurons (Fig. 3c).

The current study provides evidence that PKMζ restrains the lateral mobility of surface AMPARs. PKMζ has been reported to be upregulated after learning or LTP induction, which persists for a long time [12, 36, 37]. According to our results, the upregulation of PKMζ may reduce the lateral mobility of AMPARs, thereby affecting synaptic properties. Diffusional synaptic trapping of AMPARs is thought to be crucial for LTP [38]. A previous study demonstrated that LTP stimulus induces immobilization of AMPARs via CaMKIIα. The phosphorylation of stargazin by CaMKIIα induced the association of AMPARs with synaptic scaffolding, thereby increasing the probability of retaining synaptic AMPARs [17, 18, 22, 28]. The immobilization of AMPARs by PKMζ upregulation, found in our study, may also contribute to the synaptic stabilization of AMPARs for LTP manifestation. In our results, expression of constitutively active PKMζ T227E immobilized AMPARs in both synaptic and extrasynaptic compartments, similar to CaMKIIα in the previous study. Although ectopic over-expression was used in both studies, physiological up-regulation of CaMKIIα or PKMζ levels and their actions may occur in local subcellular environments and affect the lateral mobility of the nearby AMPARs. Particularly, movement of synaptic AMPARs at potentiated spines or extrasynaptic AMPARs at spine necks nearby the potentiated spines may be restrained to increase the probability of AMPAR at the potentiated spines. These hypotheses warrant further research.

Synaptic transmission measured by the mEPSC amplitude showed an enhancement of the size of the largest mEPSCs by PKMζ T227E and a slight tendency towards an increase in mean mEPSC, in line with prior overexpression studies [14, 35]. These results with PKMζ overexpression are in contrast to previous work with rapid postsynaptic perfusion of PKMζ, which produces a doubling of EPSC and mEPSC size within minutes of cell breakthrough [5, 11, 39]. The small effect in our results may be partly due to a portion of neurons that do not overexpress PKMζ (co-transfection efficiency ~80%), or to homeostatic changes decreasing responses at synapses without PKMζ. Another explanation would be that although the single synaptic responses did not change strongly, a population response to the same presynaptic stimulus could have increased further through processes such as activating silent synapses, given that we observed the increasing tendency in both frequency and amplitude of mEPSC. In addition to the effect on the basal synaptic transmission, a previous study has shown that PKMζ overexpression enhanced LTP [14]. Thus, as a newly synthesized plasticity-related protein (PRP), the increased PKMζ may prime neurons to respond to other events, such as formation of synaptic tags following an LTP stimulus [40]. This highlights the possibility that the upregulation of PKMζ following one type of learning may prime neurons for an experience or stimulus that follows. Intriguingly, this process may involve the regulation of lateral mobility of AMPARs seen in our results.

In our results, expression of PKMζ K98W, a dominant negative form, affected neither the lateral mobility of AMPAR nor mEPSC. Although it has no effect during the basal state, K98W may impair the changes associated with LTP when PKMζ is upregulated and in action. Thus, our result is in line with the findings that only potentiated synaptic transmission is affected by K98W and reversed by PKMζ inhibitors, whereas basal synaptic transmission is not [5, 6].

In summary, we used the single Qdot live-imaging analysis to investigate the effect of PKMζ overexpression on the lateral movement of GluA2-containing AMPARs. PKMζ overexpression decreased the lateral diffusion of GluA2-containing AMPARs at the synapse. This finding highlights a novel molecular mechanism that may underlie learning and memory. The PKMζ substrates and detailed mechanisms mediating the effect of AMPAR immobilization will be interesting to investigate in the future.

## Abbreviations

AMPA: α-amino-3-hydroxy-5-methyl-4-isoxazolepropionic acid
LTP: Long term potentiation
mEPSC: Miniature excitatory post-synaptic current
PKMζ: Protein Kinase M zeta
Qdot: Quantum-dot
ZIP: Zeta inhibitory peptide
